# Comparing the acute effects of shiftwork on mothers and fathers

**DOI:** 10.1093/occmed/kqab083

**Published:** 2021-06-24

**Authors:** P Tucker, C Leineweber, G Kecklund

**Affiliations:** 1 Stress Research Institute, Department of Psychology, Stockholm University, Stockholm, Sweden; 2 Psychology Department, Swansea University, Swansea, UK

**Keywords:** Fatigue, gender, night work, parenting, shift work, sleep, work–family conflict

## Abstract

**Background:**

Shift work may impact women more negatively than men due to the increased burden of coping with demanding work schedules while also undertaking more of the domestic chores, including childcare.

**Aims:**

To examine whether the combination of shift working and caring for children affects the sleep, fatigue and work–family conflict experienced by women more than it affects men.

**Methods:**

Using data from a survey of the Swedish working population, mixed linear regression models examined work schedule (daywork, shift work with nights, shift work without nights), gender and presence of children <13 years at home as predictors of sleep insufficiency, sleep disturbance, fatigue and work–family conflict, over up to three successive measurement occasions. Adjustments were made for age, education, full/part-time working and baseline year.

**Results:**

In fully adjusted models (*N* = 8938), shift work was associated with insufficient sleep (*P <* 0.01), disturbed sleep (*P <* 0.01), fatigue (*P <* 0.05) and work–family conflict (*P <* 0.001). Interactions in the analyses of sleep disturbance (*P <* 0.001) and work–family interference (*P <* 0.05) indicated that among participants with no children, females reported more disturbed sleep and more work–family conflict than their male counterparts, irrespective of schedule; while among participants with children, female dayworkers reported more disturbed sleep than their male counterparts, and females working shifts without nights reported more work–family interference.

**Conclusions:**

Having young children did not exacerbate negative effects of shift work, in either men or women. This may reflect high levels of gender equality and childcare provision in Sweden.

**Key learning points**

**What is already known about this subject**

- Shift work has negative effects on workers’ sleep and consequent fatigue, due in large part to the disruption of circadian rhythms. It can also increase work–family conflict as a result of the need to work unsocial hours.
- Some studies have found that shift work impacts women’s sleep, fatigue and certain other aspects of their well-being more negatively than it does men.
- Women tend to undertake more unpaid domestic work at home, including childcare, and this increased burden has been proposed as a reason for them suffering more problems with shift working.

**What this study adds**

- While shift work negatively impacts sleep, fatigue and work–family conflict, there is no evidence from the current findings that childcare exacerbates those effects, either for women or for men.
- The current study did not show that women were more negatively affected by shift work than men, with respect to sleep, fatigue or experience of work–family conflict.

**What impact this may have on practice or policy**

- Shift working parents of young children may not be at greater risk of impaired sleep, greater consequent fatigue or work–family conflict than their non-shift working counterparts.
- The findings should be interpreted in the context of the data having been obtained in Sweden, a country with high levels of gender equality and state subsidized childcare provision.

## Introduction

There is a prevailing view that women are less tolerant of shift work than men, i.e. female shift workers tend to report greater effects of shift work on sleep, fatigue, accident risk and health, although the evidence is mixed [1]. A commonly discussed source of gender-specific effects of shift work is gender roles [2]. Women in employment commonly experience a ‘double burden’ of having to cope with the demands of their job (e.g. demanding work hours), while at the same time undertaking more work at home, particularly if they have children or other adults to care for [3–5]. For example, female shift workers may be more likely than their male counterparts to forgo recovery opportunities to undertake childcare activities. Clissold *et al*. [6] reported that female nurses felt unable to use their later starting afternoon shifts as an opportunity to repay the sleep debt incurred in the night shift, opting instead to use their free time to engage in domestic chores and childcare. One of the few studies to compare males and female shift workers doing the same job found that females up to 50 years of age had shorter sleeps, were drowsier on shift, more chronically fatigued and reported poorer health, compared to their male colleagues [7]. However, the gender difference was reversed after the age of 50, which may reflect the lessening of the females’ double burden as their children grew up and eventually left home [2].

Shift work affects workers’ health, well-being and safety through three pathways: disturbance of circadian rhythms and the body clock; shortened and disturbed sleep; and disturbed family and social life [8]. These pathways are moderated by individual differences and situational factors (e.g. gender and family situation). The current study examines whether gender and the presence of children at home moderate the second and third of those pathways, i.e. whether the combination of being a shift worker and having young children at home affects the sleep, fatigue and work–family conflict experienced by women more than it affects men.

## Methods

Data were drawn from the Swedish Longitudinal Occupational Survey of Health (SLOSH). SLOSH is an open cohort survey of an approximately nationally representative sample of the working population. Follow-ups have been conducted every second year since 2006. All labour market sectors and occupations are represented, and the number of men and women is approximately equal.

Baseline data and follow-up data were drawn from five waves (i.e. there were five measurement occasions: 2010, 2012, 2014, 2016 and 2018) of SLOSH. Additional data concerning previous exposure to shift work were obtained from the 2008 wave (see exclusion criteria, described below).

**Figure 1.**
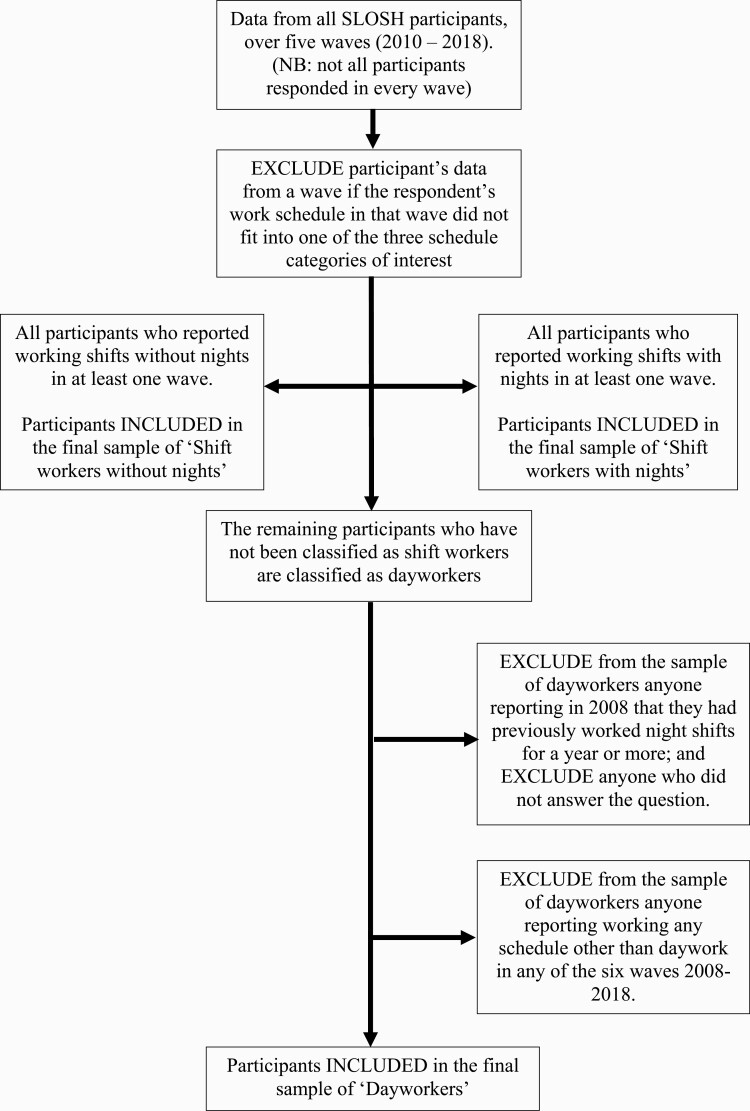
The process of categorizing participants with respect to their work schedule.

Participants were classified as being either dayworkers, shift workers who did not work nights (shift work without nights) or shift workers who did work nights (shift work with nights); see below for work schedule category definitions. The process of categorizing participants with respect to their work schedule is illustrated in Figure 1. If a participant reported in any of the five waves 2010–18 working something other than either daywork, shift work with nights or shift work without nights, their responses for that wave were excluded from the analyses. We then identified all participants who indicated being shift workers (either with or without night shifts) in any of the five waves 2010–18. When all shift workers in the dataset had been identified, the sample of dayworkers was drawn from the remainder who had not been categorized as a shift worker at any time. We sought to limit selection effects [9] by excluding responses of dayworkers with prior or subsequent exposure to shift work. This was to ensure that the shift workers were not being compared with dayworkers whose health may have been affected by prior exposure to, or a subsequent transfer to, shift work. Hence two additional exclusion criteria were applied to respondents classified as dayworkers. Participants classified as dayworkers were excluded if they had either: indicated in response to a question in the 2008 survey (the only year in which the question was asked) that they had previously worked night shifts for a year or more, or if they had not answered the question; or reported working any schedule other than daywork in any of the six waves 2008–18. The final sample after exclusions was *N* = 8938. Baseline data were based on responses from the first survey in which the respondent indicated being a dayworker/shift worker, while their outcome data were based on their responses at baseline and in the subsequent two waves (2 and 4 years later). Ethical approvals for SLOSH and the current study were obtained from the Regional Research Ethics Board in Stockholm.

Work schedule was ascertained by asking respondents about their normal working hours. Response options were *daywork (* ~*6.00 a.m.–6.00 p.m.)*, *evening work (*~*6.00 p.m.–10 p.m.)*, *nightwork (*~*6.00 p.m.–6.00 a.m.)*, *two-shift shift work*, *three-shift shift work*, *rostered work (i.e. following an ad hoc duty rota) without nightshifts*, *rostered work including nightshifts*, *discretionary/unregulated working hours* and *other*. Participants included in the current analyses were classified as undertaking either *daywork*, *shift work with nights* (either nightwork, three-shift shift work or rostered work including nights) or *shift work without nights* (two-shift shift work or rostered work without nights).

Gender was obtained from registers linked to questionnaire responses by means of the unique Swedish 10-digit personal identification numbers. Presence of children under 13 years at home was determined from a series of items asking about the number and ages of any children at home, from which participants were categorized as having either *at least one child at home under 13 years* or as *none*.

Age (at the end of the year during which the baseline questionnaire was completed) was also obtained from registers. Educational level was determined from registers indicating the respondents’ highest level of education attained, categorized as *<3 years of higher education* or *≥3 years of higher education*. Employment status was self-reported, with participants categorized as working either *full-time* or *part-time*.

Sleep disturbance, mental fatigue and sleep insufficiency were measured with items from the Karolinska Sleep Questionnaire, which assesses sleep problems experienced in the last 3 months [10,11]. Sleep disturbance was calculated as the mean of four items assessing the frequencies of: difficulty falling asleep, disturbed sleep, premature awakening and repeated awakenings; while a fifth item measured the frequency of experiencing mental fatigue (1: never; 6: always/five times a week). Sleep insufficiency was based on a single item asking whether the respondent felt that they got enough sleep (1: yes, definitely enough; 5: no, far from enough).

Work–family conflict was calculated as the mean of four items assessing the frequency with which work negatively affects life at home (1: not all; 5: nearly all the time), adapted from Fisher *et al*. [12].

Mixed linear regression models with random intercepts examined the combined effects of work schedule, gender and childcare status on sleep insufficiency, sleep disturbance, fatigue and work–family conflict across up to three measurement occasions. In each analysis, an initial model was implemented with three between-subject predictors (schedule, gender and childcare status) and one within-subject predictor (measurement occasion). A second model additionally included a set of interaction terms that were relevant for the purposes of the current enquiry. Lastly, a full model was implemented that additionally included the four covariates (age, educational level, full- /part-time status and baseline year). Interactions were explored with pairwise comparisons using Sidak adjustment for multiple comparisons. Mixed model analysis has the advantage of being flexible in handling missing data in longitudinal studies and is superior to imputation methods [13].

## Results

Descriptive statistics for the final sample, by schedule, are presented in Table 1. Adjusted means for the four main outcome variables by schedule, gender and childcare status are presented in Supplementary Material (available as Supplementary data at *Occupational Medicine* Online).

**Table 1.** Descriptive statistics for the final sample

	Daywork	Shift work with nights	Shift work without nights
	*N* (%)	*N* (%)	*N* (%)
Sex			
Male	2108 (42)	819 (46)	651 (30)
Female	2878 (58)	944 (53)	1538 (70)
Childcare status			
No child <13 years at home	3659 (73)	1362 (77)	1725 (79)
Child <13 years at home	1327 (27)	401 (23)	464 (21)
Baseline year			
2010	3929 (79)	556 (31)	724 (33)
2012	701 (14)	181 (10)	227 (10)
2014	200 (4)	688 (39)	722 (33)
2016	110 (2)	225 (13)	337 (15)
2018	46 (1)	113 (6)	179 (8)
Educational level			
<3 years of higher education	2809 (56)	1218 (69)	1649 (75)
≥3 years of higher education	2176 (44)	543 (31)	539 (25)
Employment			
Part-time	978 (20)	538 (31)	684 (32)
Full-time	3932 (80)	1214 (69)	1483 (68)
	Mean (SE)	Mean (SE)	Mean (SE)
Age (years)	50.24 (0.14)	48.34 (0.25)	49.94 (0.22)

The number of responses in the waves from 2008 to 2018 were 11 441, 11 525, 9880, 20 316, 19 360 and 17 841 (response rates 62, 56, 57, 53, 51 and 48%).

The main findings are summarized below, and a full account is presented in Supplementary Material (available as Supplementary data at *Occupational Medicine* Online). Table 2 presents the results from the third (final) models that included interaction terms and covariates.

**Table 2.** *F* values from mixed linear regression final models that included interaction terms and covariates

	Sleep insufficiency	Sleep disturbance	Fatigue	Work–family conflict
Schedule	5.36**	6.54**	4.20*	17.79***
Childcare	11.47**	0.80	0.74	2.26
Gender	1.79	51.59***	104.53***	56.37***
Measurement occasion (MO)	19.32***	0.42	23.10***	9.40 ***
Schedule * MO	1.76	1.29	5.29***	1.82
Schedule * Childcare	1.51	0.41	0.54	3.03*
Schedule * Gender	5.28**	2.95	0.34	4.12*
Schedule * Childcare * MO	1.74	1.08	0.39	0.64
Schedule * Gender * MO	2.43*	1.06	1.68	1.03
Schedule * Childcare * Gender	1.89	5.36**	1.24	3.23*
Schedule * Childcare * Gender * MO	1.33	1.49	0.50	0.40
Age	100.54***	21.39***	109.4***	17.16***
Education	7.29**	0.19	13.30***	30.00***
Full-time	25.59***	8.35**	5.64*	68.23***
Baseline year	0.83	1.51	4.61**	2.14

*P < 0.05; **P < 0.01; ***P < 0.001.

In the analysis of sleep insufficiency (first model), greater sleep insufficiency was predicted by shift work without nights (*b* = 0.083, 95% confidence intervals (CIs) 0.041–0.125, *t*(8905.51) = 3.90, *P <* 0.001), shift work with nights (*b* = 0.080, 95% CI 0.035–0.125, *t*(8941.24) = 3.49, *P <* 0.001), presence of young children at home (*b* = 0.229, 95% CI 0.190–0.269, *t*(8901.05) = 11.26, *P <* 0.001) and female gender (*b* = 0.077, 95% CI 0.042–0.113, *t*(8927.79) = 4.32, *P <* 0.001). In the second and third models, there were no significant interactions involving the combination of schedule, gender and childcare status.

**Figure 2.**
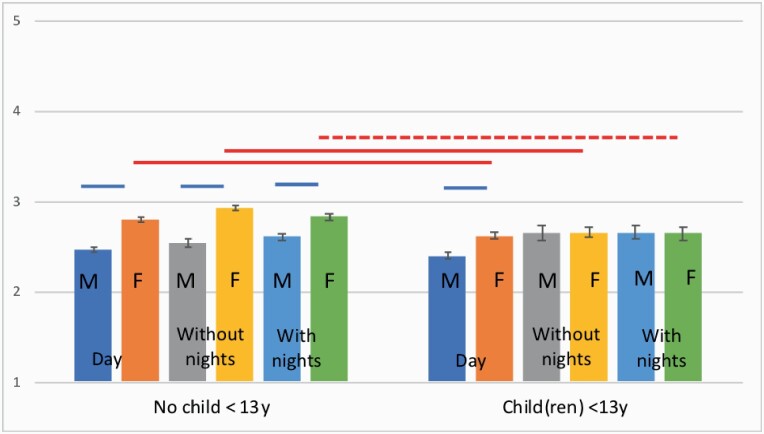
Interaction between schedule, gender and childcare status in the measurement of sleep disturbance (unadjusted means). Red horizontal lines represent pairwise comparisons between subgroups with- and without young children at home; blue horizontal lines represent male and female subgroups; solid horizontal lines represent significant differences (*P <* 0.01), dashed horizontal lines represent significant differences (*P <* 0.05),

In the analysis of sleep disturbance (first model), greater sleep disturbance was predicted by shift work without nights (*b* = 0.112, 95% CI 0.062–0.162, *t*(8843.23) = 4.37, *P <* 0.001), shift work with nights (*b* = 0.095, 95% CI 0.040–0.145, *t*(8804.77) = 3.44, *P <* 0.01), *absence* of young children at home (*b* = −0.117, 95% CI −0.165 to −0.069, *t*(8661.57) = −4.81, *P <* 0.001) and female gender (*b* = 0.263, 95% CI 0.221–0.306, *t*(8698.70) = 12.21, *P <* 0.001). In the second model, there was a significant three-way interaction between schedule, gender and childcare status (*F*(3,9490.04) = 5.84, *P <* 0.01; see Figure 2). Among respondents without young children, females in all schedule groups reported more sleep disturbance than their male counterparts; whereas among respondents with young children at home, there was only a significant gender difference for dayworkers. The same interaction was observed in the third model.

In the analysis of fatigue (first model), greater fatigue was predicted by shift work without nights (*b* = 0.121, 95% CI 0.062–0.180, *t*(8806.80) = 4.03, *P <* 0.001), presence of young children at home (*b* = 0.158, 95% CI 0.103–0.214, *t*(8559.83) = −5.58, *P <* 0.001) and female gender (*b* = 0.384, 95% CI 0.335–0.434, *t*(8605.49) = 15.30, *P <* 0.001). In the second and third models, there were no significant interactions involving the combination of schedule, gender and childcare status.

**Figure 3.**
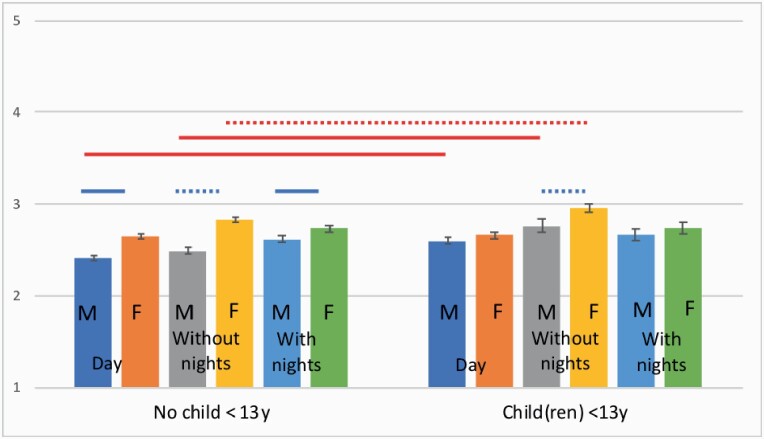
Interaction between schedule, gender and childcare status in the measurement of work–family interference (unadjusted means). Red horizontal lines represent pairwise comparisons between subgroups with- and without young children at home; blue horizontal lines represent male and female subgroups; solid horizontal lines represent significant differences (*P <* 0.01), dashed horizontal lines represent significant differences (*P <* 0.05).

In the analysis of work–family conflict (first model), greater work–family was predicted by shift work without nights (*b* = 0.166, 95% CI 0.115–0.206, *t*(8808.22) = 7.24, *P <* 0.001), shift work with nights (*b* = 0.127, 95% CI 0.079–0.176, *t*(8755.98) = 5.16, *P <* 0.001), presence of young children at home (*b* = 0.096, 95% CI 0.053–0.139, *t*(8473.36) = 4.41, *P <* 0.001) and female gender (*b* = 0.189, 95% CI 0.151–0.227, *t*(8641.10) = 9.78, *P <* 0.001). In the second model, there was a three-way interaction between schedule, gender and childcare status (*F*(3,9469.27) = 3.48, *P <* 0.05; see Figure 3). Among respondents without young children, females in all schedule groups reported more work–family interference than their male counterparts; whereas among respondents with young children at home, there was only a significant gender difference for those working shifts without nights. The same interaction was observed in the third model.

## Discussion

Shift working was associated with shorter and more disturbed sleep, greater fatigue and greater work–family conflict, in accordance with the model of Barton *et al*. [8]. However, the results provided no support for the contention that having young children exacerbates these effects of shift work, either in men or in women.

Females were more likely than males to report insufficient sleep, disturbed sleep, fatigue and work–family conflict. However, there was little indication that females were more negatively affected by shift work than males, irrespective of childcare status. While this is inconsistent with the prevailing view of women being less tolerant of shift work [1], it is consistent with previous findings of limited gender-specific effects of shift work on health found in SLOSH [14,15]. Moreover, recent evidence from meta-analyses suggest that gender-specific effects of shift work may not be as pervasive as previously suggested, at least with respect to sleep [16,17], cardiovascular disease [18] and cancer [19]. Similarly, a recent large-scale study found no gender-specific effects of shift work on accident risk [20]. However, other recent meta-analyses have shown that the links between shift work and metabolic disorders [21,22], and between shift work and mental ill-heath [23], are stronger in women than in men.

There was little or no indication of an accumulation of negative effects across successive measurement occasions. This is perhaps unsurprising, as the anticipated effects were likely to be relatively acute. While the presence of children in combination with a demanding schedule could be expected to affect sleep and work–family conflict, there is little reason to anticipate these problems getting worse with prolonged exposure. Indeed, the opposite could be argued, on the assumption that the effects might diminish as children get older and become more independent. A sensitivity analysis (not reported above) found that changing the cut-off age for the definition of young children from 13 years to 6 years made no substantive difference to the patterns of results obtained.

The strengths of the current study include a prospective design based on multiple repeated measures that consider the time-varying nature of the studied effects. The analytic approach accounts for missing values, making it possible to draw upon a large, heterogeneous and broadly representative sample of the working population in Sweden.

The current analysis sought to limit selection effects by excluding from the day working sample any respondents with a history of working night shifts. This was to ensure that shift workers were not being compared with dayworkers whose health may have been impaired due to previous exposure to nightwork, as a result of which they may have transferred into daywork [9]. However, selection effects cannot be completely ruled out. It was only possible to exclude dayworkers reporting prior exposure to *nightwork* in 2008, but not those who worked shifts that did not include nightwork.

It could be suggested that the failure to find the expected pattern of results reflect insensitivity of the outcome measures, particularly as two of the four (sleep insufficiency and fatigue) were based on single-item measures. However, counter to that suggestion, it is notable that all four outcome measures *did* show negative effects of shift work; but that these effects were not exacerbated by either gender, childcare status or a combination of the two. Nevertheless, the majority of measures were self-report, which introduces the possibility of reporting bias or recall error, as well as bias due to common-method variance [24]. The measure of shift work exposure was crude, with no possibility to account for variations in schedules such as the sequence of shifts, intensity of nightwork, etc. It is possible that female shift workers, who primarily work in healthcare settings where self-scheduling is common (in Sweden), had more possibilities than male shift workers to adapt their schedules to having young children, e.g. by limiting nightwork. The measure of work–family interference does not focus specifically on children, which limits its sensitivity in the current context. The measure of part-time working lacked a definition in terms of weekly work hours which may limit its sensitivity, given that part-time working in Sweden can involve working as much as 35 hours per week. While the analysis excluded dayworkers who subsequently changed to shift work during follow-up, it remains a possibility that some shift workers may have transferred out of shift work during follow-up, which could have reduced the apparent effect of shift work.

Female and male shift workers tend to work in different occupational sectors and so it is often argued that gender comparisons of the effects of shift work are confounded by occupation. The current study did not adjust for occupation, as we have found that, in the SLOSH sample at least, differences in the occupational characteristics of female and male dominated occupations do not account for gender differences in associations between shift work and sleep disturbance or health [15].

It could be argued that with such a relatively large sample, the statistical power of the current study design may have contributed to the finding of effects that, though relatively small, were statistically significant. It is notable, however, that the observed significant main effects were in accordance with previous findings of associations between shift work and sleep/fatigue [25]; between shift work and work–family conflict [26]; between gender and sleep/fatigue [27]; and between gender and work–family conflict [3,4].

The current findings offer no basis for treating women and men differently with respect to work scheduling or the provision of support for those working non-standard hours. However, while we failed to observe the predicted pattern of interaction between schedule, gender and childcare status, it should be noted that these data were collected in Sweden where there is universal subsidized childcare for children from a very early age, which may serve to mitigate the double burden of demanding work hours and childcare responsibilities. Moreover, Sweden has high levels of gender equality which may mean that the added burden of childcare is relatively evenly distributed between the sexes, such that neither experiences excessive disruption of sleep and family life. Thus, caution is advised when attempting to extrapolate from these findings to other countries with different socio-political contexts. An alternative explanation for the absence of predicted interactions is that the women in this study did experience greater strain as result of a higher ‘double burden’, compared to the men, but that they were able to adopt effective strategies to cope with or compensate for it [28].

In conclusion, shift work, female gender and presence of young children at home were all associated with negative effects on sleep, fatigue and work–family conflict. However, contrary to expectations, neither female gender nor presence of young children exacerbated the effects of shift work. It remains to be seen whether these three factors act synergistically upon other more chronic aspects of health.

## Supplementary Material

kqab083_suppl_Supplementary_Material
