# Supplementary material for: Comparing the acute effects of shiftwork on mothers and fathers
Source: Occup Med (Lond). 2021 Jun 24;71(9):414–21. doi: 10.1093/occmed/kqab083 (PMC8703007; doi:10.1093/occmed/kqab083)
Supplement: kqab083_suppl_Supplementary_Material [file kqab083_suppl_supplementary_material.doc]

**Online supplementary table. Adjusted means of the four main outcome variables by childcare status, schedule and gender**

|  |  |  | Sleep insufficiency | | Sleep disturbance | | Fatigue | | Work-family conflict | | |
| --- | --- | --- | --- | --- | --- | --- | --- | --- | --- | --- | --- |
|  |  |  | Mean | SE | Mean | SE | Mean | SE | Mean | SE |  |
| No child < 13 years at home | Daywork | Male | 2.24 | 0.03 | 2.44 | 0.04 | 2.31 | 0.04 | 2.39 | 0.03 |  |
|  |  | Female | 2.37 | 0.03 | 2.79 | 0.03 | 2.72 | 0.04 | 2.63 | 0.03 |  |
|  | Shift work without nights | Male | 2.40 | 0.04 | 2.50 | 0.05 | 2.32 | 0.06 | 2.45 | 0.05 |  |
|  |  | Female | 2.47 | 0.03 | 2.91 | 0.03 | 2.79 | 0.04 | 2.84 | 0.03 |  |
|  | Shift work with nights | Male | 2.38 | 0.04 | 2.57 | 0.04 | 2.33 | 0.05 | 2.57 | 0.04 |  |
|  |  | Female | 2.43 | 0.03 | 2.81 | 0.04 | 2.59 | 0.05 | 2.73 | 0.04 |  |
| Child < 13 years at home | Daywork | Male | 2.40 | 0.04 | 2.43 | 0.05 | 2.28 | 0.05 | 2.50 | 0.04 |  |
|  |  | Female | 2.50 | 0.04 | 2.67 | 0.04 | 2.67 | 0.05 | 2.58 | 0.04 |  |
|  | Shift work without nights | Male | 2.49 | 0.07 | 2.66 | 0.08 | 2.44 | 0.10 | 2.66 | 0.07 |  |
|  |  | Female | 2.50 | 0.05 | 2.71 | 0.06 | 2.71 | 0.07 | 2.91 | 0.05 |  |
|  | Shift work with nights | Male | 2.57 | 0.06 | 2.67 | 0.08 | 2.18 | 0.09 | 2.56 | 0.07 |  |
|  |  | Female | 2.40 | 0.06 | 2.69 | 0.08 | 2.59 | 0.09 | 2.67 | 0.07 |  |

**Online supplementary material**

**Full description of the results of the main analysis**

In the analysis of sleep insufficiency, in the first model, greater sleep insufficiency was predicted by shift work without nights (b = 0.083, 95% confidence intervals (CI) 0.041 to 0.125, t(8905.51) = 3.90, *P <* .001), shift work with nights (b = 0.080, 95% CI 0.035 to 0.125, t(8941.24) = 3.49, *P <* .001), presence of young children at home (b = 0.229, 95% CI 0.190 to 0.269, t(8901.05) = 11.26, *P <* .001) and female gender (b = 0.077, 95% CI 0.042 to 0.113, t(8927.79) = 4.32, *P <* .001). There was also a significant effect of measurement occasion, with sleep insufficiency decreasing over time, i.e. sleep duration increased overtime (b_time1_ = 0.106, 95% CI 0.084 to 0.128, t(7348.38) = 9.50, *P <* .001); b_time2_ = 0.058, 95% CI 0.037 to 0.080, t(11241.28) = 5.37, *P <* .001; b_time3_=0 [reference]). The second model, in which interaction terms were added, significantly improved the fit over the first model (χ^2^_Change_ (29) = 62.46, *P* < .01). The main effect of female gender became non-significant. There was a significant two-way interaction between schedule and gender (F(2,9882.85) = 6.50, *P <* .01) and a three-way interaction between schedule, gender and measurement occasion (F(6,11008.85) = 2.45, *P <* .05). Male dayworkers reported less sleep insufficiency (i.e. more sleep) than their female counterparts (and less than all shift worker groups, male and female), particularly on the first two measurement occasions (mean differences: time 1 = .156, *P <* .001; time 2 = .125, *P* < .001; time 3 = .081, *P <* .05). There were no significant interactions involving the combination of schedule, gender and childcare status. The fully adjusted third model significantly improved the fit over the second model (χ^2^_Change_ (7) = 156.52, *P* < .01) while producing the same pattern of effects as the second model (see Table 2).

In the analysis of sleep disturbance, in the first model, greater sleep disturbance was predicted by shift work without nights (b = 0.112, 95% CI 0.062 to 0.162, t(8843.23) = 4.37, *P <* .001), shift work with nights (b = 0.095, 95% CI 0.040 to 0.145, t(8804.77) = 3.44, *P <* .01), *absence* of young children at home (b = -0.117, 95% CI -0.165 to -0.069, t(8661.57) = -4.81, *P <* .001) and female gender (b = 0.263, 95% CI 0.221 to 0.306, t(8698.70) = 12.21, *P <* .001). There was also a significant effect of measurement occasion, with a dip in sleep disturbance at the second measurement occasion (b_time1_ = -0.018, 95% CI -0.042 to 0.007, t(6283.91) = -1.39, NS); b_time2_ = -0.033, 95% CI -0.057 to -0.008, t(9818.62) = -2.65, *P <* .01; b_time3_=0 [reference]). The second model significantly improved the fit over the first model (χ^2^_Change_ (29) = 53.06, *P* < .01). There was a significant two-way interaction between schedule and gender (F(2,9654.97) = 3.10, *P <* .05) and a three-way interaction between schedule, gender and childcare status (F(3,9490.04) = 5.84, *P <* .01; see Figure 2). Among respondents without young children, females in all schedule groups reported more sleep disturbance than their male counterparts (mean differences between females and males: daywork = .329, *P <* .001; shift work without nights = .388, *P <* .001; shift work with nights = .223, *P <* .001); whereas, among respondents with young children at home, there was only a significant gender difference for dayworkers (mean difference = .216, *P <* .001). The fully adjusted third model significantly improved the fit over the second model (χ^2^_Change_ (7) = 33.85, *P* < .01). The main effect of childcare status became non-significant but otherwise the pattern of effects was similar to the second model (see Table 2).

In the analysis of fatigue, in the first model, greater fatigue was predicted by shift work without nights (b = 0.121, 95% CI 0.062 to 0.180, t(8806.80) = 4.03, *P <* .001), presence of young children at home (b = 0.158, 95% CI 0.103 to 0.214, t(8559.83) = -5.58, *P <* .001) and female gender (b = 0.384, 95% CI 0.335 to 0.434, t(8605.49) = 15.30, *P <* .001). There was also a significant effect of measurement occasion, with fatigue peaking at the second measurement occasion (b_time1_ = -0.030, 95% CI -0.064 to 0.003, t(9970.53) = -1.77, NS); b_time2_ = 0.134, 95% CI 0.100 to 0.169, t(9970.53) = 7.69, *P <* .001; b_time3_=0 [reference]). The second model significantly improved the fit over the first model (χ^2^_Change_ (29) = 49.20, *P* < .05). There was a significant two-way interaction between schedule and measurement occasion (F(4,9910.30) = 5.45, *P <* .001). For dayworkers and those working shift without nights, fatigue peaked at the second measurement occasion (mean change from first to second, and second to third measurement occasions: daywork = .209, *P <* .001 and -.165, *P <* .001; shift work without nights = .159, *P <* .001 and -.235, *P <* .001); whereas for those working shifts with nights, there was no significant change between successive measurement occasions (mean changes = .013, NS and -.27, NS). There were no significant interactions involving the combination of schedule, gender and childcare status. The fully adjusted third model significantly improved the fit over the second model (χ^2^_Change_ (7) = 158.91, *P* < .01). The main effect of childcare status became non-significant, but otherwise the pattern of effects was similar to the second model (see Table 2).

For the analysis of work-family conflict, in the first model, greater work-family was predicted by shift work without nights (b = 0.166, 95% CI 0.115 to 0.206, t(8808.22) = 7.24, *P <* .001), shift work with nights (b = 0.127, 95% CI 0.079 to 0.176, t(8755.98) = 5.16, *P <* .001), presence of young children at home (b = 0.096, 95% CI 0.053 to 0.139, t(8473.36) = 4.41, *P <* .001) and female gender (b = 0.189, 95% CI 0.151 to 0.227, t(8641.10) = 9.78, *P <* .001). There was also a significant effect of measurement occasion, with a dip in work-family conflict at the second measurement occasion (b_time1_ = -0.021, 95% CI -0.002 to 0.045, t(6365.07) = 1.79, NS); b_time2_ = -0.044, 95% CI -0.067 to -0.021, t(9792.88) = -3.71, *P <* .001; b_time3_=0 [reference]). The second model significantly improved the fit over the first model (χ^2^_Change_ (29) = 57.27, *P* < .01). There was a three-way interaction between schedule, gender and childcare status (F(3,9469.27) = 3.48, *P <* .05; see Figure 3). Among respondents without young children, females in all schedule groups reported more work-family interference than their male counterparts (mean differences between females and males: daywork = .235, *P <* .001; shift work without nights = .337, *P <* .001; shift work with nights = .115, *P <* .05); whereas, among respondents with young children at home, there was only a significant gender difference for those working shifts without nights (mean difference = .199, *P <* .05). The fully adjusted third model significantly improved the fit over the second model (χ^2^_Change_ (7) = 140.73, *P* < .01). The main effect of childcare status became non-significant. Also, the two-way interactions between schedule and childcare status, and between schedule and gender, became significant. Otherwise, the pattern of effects was similar to the second model (see Table 2).
